# Germinality does not necessarily define mAb expression and thermal stability

**DOI:** 10.1007/s00253-019-09998-3

**Published:** 2019-07-26

**Authors:** Linda Schwaigerlehner, Patrick Mayrhofer, Matthias Diem, Willibald Steinfellner, Emma Fenech, Chris Oostenbrink, Renate Kunert

**Affiliations:** 1 Department of Biotechnology, University of Natural Resources and Life Sciences, Muthgasse 18, 1190 Vienna, Austria; 2 Department of Material Sciences and Process Engineering, University of Natural Resources and Life Sciences, Muthgasse 18, 1190 Vienna, Austria; 3 Ludwig Institute for Cancer Research, University of Oxford, Oxford, UK

**Keywords:** Recombinant antibody production, Difficult to express, Mammalian expression system, Secretory pathway, Germinalization

## Abstract

The production potential of recombinant monoclonal antibody (mAb) expressing cell lines depends, among other factors, on the intrinsic antibody structure determined by the amino acid sequence. In this study, we investigated the influence of somatic mutations in the V(D)J sequence of four individual, mature model mAbs on the expression potential. Therefore, we defined four couples, each consisting of one naturally occurring mAb (2G12, Ustekinumab, 4B3, and 2F5) and the corresponding germline-derived cognate mAb (353/11, 554/12, 136/63, and 236/14). For all eight mAb variants, recombinant Chinese hamster ovary (CHO) cell lines were developed with mAbs expressed from a defined chromosomal locus. The presented workflow investigates critical parameters including productivity, intra- and extracellular product profile, *XBP1* splicing, thermal stability, and *in silico* hydrophobicity. Significant differences in productivity were even observed between the germline-derived mAbs which did not undergo somatic mutagenesis. Accordingly, back-to-germline mutations of mature mAbs are not necessarily reflecting improved expression and stability but indicate opportunities and limits of mAb engineering. From our studies, we conclude that germinalization represents a potential to improve mAb properties depending on the antibody’s germline family, highlighting the fact that mAbs should be treated individually.

**Electronic supplementary material:**

The online version of this article (10.1007/s00253-019-09998-3) contains supplementary material, which is available to authorized users.

## Introduction

Antibodies are among the key components involved in the adaptive immune system with a significant increase of monoclonal antibodies (mAbs) in therapeutic application (Kaplon and Reichert 2018). The diversity of antibodies is generated during plasma cell maturation by V(D)J recombination, insertions at the rearrangement sites, and somatic (hyper)mutation (Kim et al. 1981; Maizels 2005).

Antibody properties, such as productivity/availability in the plasma and thermal stability, are influenced by the choice of the V_H_ and V_L_ families (Ewert et al. 2003). Moreover, preferential combinations for V_H_/V_L_ pairing affect the antibody properties (Jayaram et al. 2012; Chen et al. 2015) and a tighter and more compact packing of the V_H_/V_L_ allows facilitated expression (Plückthun et al. 1996). Another important factor is the length of the CDR-H3 loop, which has previously been found to influence the expression properties (Pybus et al. 2014).

Here, we present a comparison of four affinity matured antibodies and their corresponding germline variants as related couples in terms of expression potential and thermal stability properties. We adapted the recombinase-mediated cassette exchange (RMCE) concept in CHO K1 cells to allow insertion of the gene of interest into a pre-defined chromosomal locus with invariable gene copy numbers (Schlake and Bode 1994; Seibler et al. 1998). Such single-copy recombinant cell lines are defined as isogenic (Mayrhofer et al. 2014) and enable the investigation of the expression of different mAbs during cell propagation. We analyzed intra- and extracellular product accumulation. As IgGs are secretory proteins, we considered that insufficient secretion may be a result of accumulation in the ER lumen, which could induce ER stress and hence activate the unfolded protein response (UPR). The UPR consists of three signaling pathways, the most conserved of which is the IRE1 branch. Upon activation, IRE1 oligomerizes and splices *XBP1* mRNA (reviewed in Ron and Walter 2007) and therefore ER stress was monitored using an *XBP1* splicing assay (Lin et al. 2007). The purified mAbs were also analyzed for thermal stability and additionally we identified aggregation-prone regions on the antibody binding site as well as hydropathic regions. To evaluate the obtained expression data with a complementary strategy, we expressed the same mAbs in scFv-Fc format transiently.

As mAb models, we focused on anti-HIV1 antibodies, as they show a higher somatic mutation rate compared to other IgGs (Scheid et al. 2009; Xiao et al. 2009a; Xiao et al. 2009b). 2G12 (Buchacher et al. 1994; Trkola et al. 1996; Kunert et al. 1998), 4B3 (Buchacher et al. 1994), and 2F5 (Buchacher et al. 1994; Purtscher et al. 1994; Kunert et al. 1998) were selected as model antibodies. The three chosen anti-HIV1 antibodies carry 30-41 V_H_ gene somatic mutations, whereas most mature human antibodies exhibit 15-20 V_H_ gene mutations (Tiller et al. 2007; Mayrhofer and Kunert 2018). In order to ensure that an effect on expression and thermal stability is not attributable to the excessive somatic hypermutation found in anti-HIV1 antibodies or the accumulation of somatic mutations in the framework region (Klein et al. 2013), germinalization is also evaluated in one therapeutic antibody. We chose Ustekinumab (Bartlett and Tyring 2008; Leonardi et al. 2008; Papp et al. 2008), a therapeutic antibody that exhibits 17 V_H_ gene somatic mutations (Table 1).

**Table 1:**
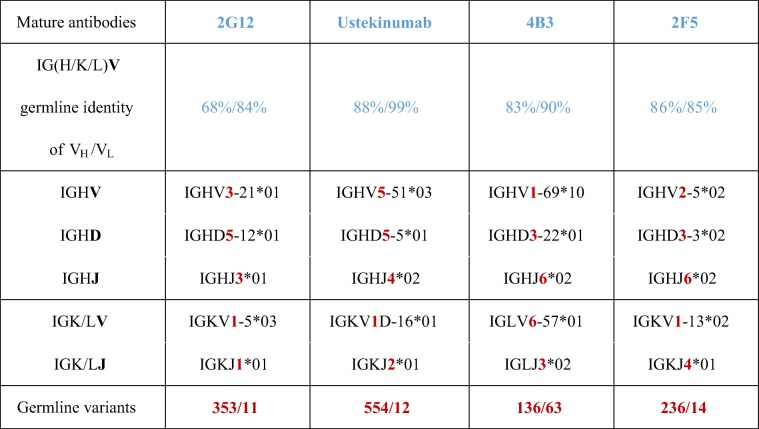
Sequence identity to the closest human germline sequence (germinality). Respective antibody germline variants were designed by combination of V, (D), and J segments.

## Materials and methods

### MAbs and recombinant cell lines

#### Design of germline variants

A panel of four mature naturally occurring human mAbs was defined: 2G12, Ustekinumab, 4B3, and 2F5. This antibody set includes three anti-HIV1 antibodies directed against gp120 or gp41, as well as the therapeutic antibody, Ustekinumab, which is directed against IL12/23. For each mature antibody, a germline-derived cognate mAb was designed by combining germline segments (V, (D), and J), nearest related to the mature antibodies. The non-binding germline variants were designated in the numbers of the chosen VDJ/VJ gene segments: 353/11, 554/12, 136/63, and 236/14 (Table 1). Germline identity of mature antibodies varies throughout all four variants and is highest for the therapeutic antibody Ustekinumab. The sequence alignments of the mature and germline antibodies are shown in Figure S1 in the supplementary material.

#### Development of isogenic IgG-producing cell lines

For generation of IgG-producing cell lines, the host cell line, CHO RMCE I3 (CHO K1: S1/0.3/I3 cells, described in Fig. S2), was transfected with polyethylenimine (PEI; 25 kDa, linear; Polysciences, no. 23966) and 8 μg DNA/10^6^ cells in ProCHO5 medium (Lonza, no. 12-766Q) supplemented with 4 mM l-alanyl glutamine (Merck Millipore, no. K0302) and 15 mg/L phenol red (Sigma-Aldrich, no. P0290). Four hours post transfection, the medium was changed to CD CHO (Life Technologies, no. 10743029) supplemented with 4 mM glutamine and 15 mg/L phenol red. Limiting dilution cloning of the transfection pool was performed in 384-well plates under selection pressure by ganciclovir (GCV) (Chakraborty et al. 2013). A concentration of 20 μM GCV (Sigma-Aldrich, no. G2536) was applied 3 days post transfection and reduced to 10 μM GCV after 10 days and 2 μM GCV after 24 days. Screening was done by a standard gamma-gamma sandwich enzyme-linked immunosorbent assay (ELISA) and homogeneity was proven by flow cytometry. Genomic polymerase chain reaction (PCR) was applied to confirm the exchange of the parental cassette by the antibody cassette.

#### Cloning of scFv-Fc antibodies and transient transfection in HEK293-6E

The single-chain fragment variable-fragment crystallizable antibody (scFv-Fc) constructs were generated by overlap-extension PCR according to Mayrhofer and Kunert (2017). Transient transfections were performed as described in Schwaigerlehner et al. (2018).

### Bioprocessing in semi-continuous perfusion experiments

Perfusion mode enables the comparison of cell lines in a steady state under semi-constant environment (Villiger-Oberbek et al. 2015). For the semi-continuous perfusion, all CHO cell lines were seeded at 5 × 10^6^ cells/mL in CD CHO media supplemented with 4 mM l-glutamine (Roth, no. 9183.1), 15 mg/L phenol red, and either 0.5 mg/mL G418 (Biochrom, no. A2912) for the host cell line or 2 μM GCV for antibody-producing cell lines. Cultivation of 10 mL cell suspension was done in 50-mL reactor tubes (Corning, no. 431720) and media was exchanged daily by centrifugation at 200×*g* for 10 min. In these quasi steady-state conditions, the cell concentration characteristically reaches a plateau after day 6 (Reinhart et al. 2018). IgG-producing cell lines were cultivated in triplicates and as a control, the host cell line CHO RMCE I3 was included in each experiment. Cell numbers in suspension were determined by Vi-CELL XR (Beckman Coulter) and cell viability was determined using trypan blue (Sigma-Aldrich, no. T8154) dye exclusion. IgG concentrations were determined by Bio-Layer Interferometry using an Octet™ QK (Pall) equipped with protein A biosensors as described in Reinhart et al. (2015).

### Flow cytometry

CHO cells were fixed with ice-cold 70% (*v/v*) ethanol on day 1 and day 10 of semi-continuous perfusion experiment. For intracellular HC and LC product analysis, 10^6^ cells were stained with biotinylated polyclonal anti-human gamma- (Life technologies, no. A18821), anti-human kappa- (Antibodies-online, no. ABIN375958), or anti-human lambda-chain antibody (Novus Biologicals, no. NB100-62142) and conjugated with streptavidin-Alexa Fluor 647 (Life Technologies, no. A21244) according to the protocol published in Reinhart et al. (2014). Measurement of 10,000 events per sample was performed with a Gallios flow cytometer (Beckman Coulter). Gating was done based on forward and side scatter properties using CHO RMCE I3 as a negative control. Intracellular HC and LC content was analyzed by FL-6 laser channel using Kaluza Analysis Software (Beckman Coulter). Median fluorescence intensities (MFIs) were compared of three replicates for each antibody variant.

### SDS PAGE and Western blot

Cell lysate of 2 × 10^6^ cells of semi-continuous perfusion samples after 4 days was extracted by 0.4 mL radioimmunoprecipitation assay (RIPA) buffer (Sigma-Aldrich, no. R0278) with addition of cOmplete Mini EDTA-free Protease Inhibitor Cocktail (Roche, no. 11836170001) according to the manufacturer's instructions. Whole cell lysates (6 μg total protein) and culture supernatant pools (0.3 μg total IgG) were diluted in NuPAGE LDS 4× sample buffer (Life Technologies, no. NP0008). As a molecular-weight marker, PAGEruler prestained protein ladder (Thermo Scientific, no. 26616) was used. Samples were electrophoretically separated on NuPAGE Novex 4–12% Bis-Tris gels (Life Technologies, no. NP0322) run in MOPS buffer under non-reducing conditions and transferred on a Roti-PVDF membrane, pore size 0.45 μm (Roth, no. T830.1) using NuPAGE Transfer Buffer (Life technologies, no. NP0006). Analysis was done with polyclonal anti-human gamma-chain peroxidase antibody (Invitrogen, no. 62-8420), anti-human kappa-chain (bound and free) peroxidase antibody (Sigma, no. A7164), or anti-human lambda-chain (bound and free) peroxidase antibody (Sigma, no. A5175). For visualization of chemiluminescent, SuperSignal West Pico Chemiluminescent Substrate (Thermo Scientific, no. PI34087) was used.

### *XBP1* splicing

Tunicamycin (TM) is a drug typically used to induce ER stress which activates the UPR (Walter and Ron 2011). CHO K1 cells were treated with 0.5 μg/mL and 1 μg/mL TM (Sigma-Aldrich, no.T7765) for 2 h, 4 h, and 6 h. RNA of 10,000 cells was isolated using the RNeasy Mini Kit (Qiagen, no. 74104) according to the manufacturer’s instructions. Isolated RNA (200 ng) was used for reverse transcription (RT) with oligo dT and hexamer primers using QuantiTect Reverse Transcription Kit (Qiagen, no. 205311). For determination of *XBP1* splicing (Lin et al. 2007), PCR amplification was performed with XBP1_CHO_fw (5′-TTGAGAGAGAAAACTCATGGC-3′) and rev (5′-GGGTCCAACTTGTCCAGAATGC-3′) primers in the conditions: 95 °C/5 min, 35× (95 °C/1 min, 58 °C/30 s, 72 °C/30 s) 72 °C/5 min. Separation of the PCR product was achieved on a 2.5% agarose gel using TAE buffer. Visualization of the band was enabled by peqGREEN DNA/RNA Dye (VWR, 732-3196) and 1 Kb Plus DNA Ladder (Thermo Fisher Scientific, no. 10787018) was used as marker. Intensity of the resulting bands was evaluated with the ImageJ software (Schneider et al. 2012).

### Differential scanning calorimetry

The IgGs were purified by protein A chromatography using a 1-mL pre-packed HiTrap™ MabSelect SuRe™ column (GE Healthcare, no. 29-0491-04) on an ÄKTA start system (GE Healthcare) according to the manufacturer’s recommendations. IgG samples were re-buffered by PD MidiTrap G25 units (GE Healthcare, no. 17-0851-01) in 30 mM phosphate buffer, 150 mM NaCl, and pH 6 and adjusted to 2–3 μM. Heat energy uptake was measured by a VP-DSC MicroCal LLC equipment (GE Healthcare). The heat capacity was monitored between 20 and 100 °C applying a scan rate of 1 °C/min. The baseline correction was performed by subtraction of a re-scan of the unfolded protein and the result was fitted with the Origin 7.0 software (OriginLab, Northampton, MA). For fitting, a non-two-state unfolding model was applied.

### Hydropathy and spatial aggregation propensity

The distribution of hydrophilic and hydrophobic residues of V_H_ and V_L_ was determined by using the ProtScale tool on the ExPASy server (Gasteiger et al. 2005). The amino acid hydrophobicity scale was defined according to Kyte and Doolittle (1982) and parameters were set to window size 9, relative weight 100%, linear model, and no normalization.

The V_H_ and V_L_ of all eight antibody variants were modeled by PIGSPro (Marcatili et al. 2008). Aggregation-prone regions of the paratopes were compared by the spatial aggregation propensity (SAP) (Chennamsetty et al. 2009; Chennamsetty et al. 2010). The SAP identifies hydrophobic patches on the three-dimensional structure of a protein by taking the hydrophobicity of neighboring residues into account. The SAP was calculated applying the Kyte and Doolittle hydrophobicity scale and a SAP radius of 0.5 nm. H atoms were excluded from the calculations. The results were visualized using PyMOL Molecular Graphics System, version 1.7.4.5., Schrödinger, LLC.

## Results

### Expression potential of model antibody variants in semi-continuous perfusion cultures

**Fig. 1:**
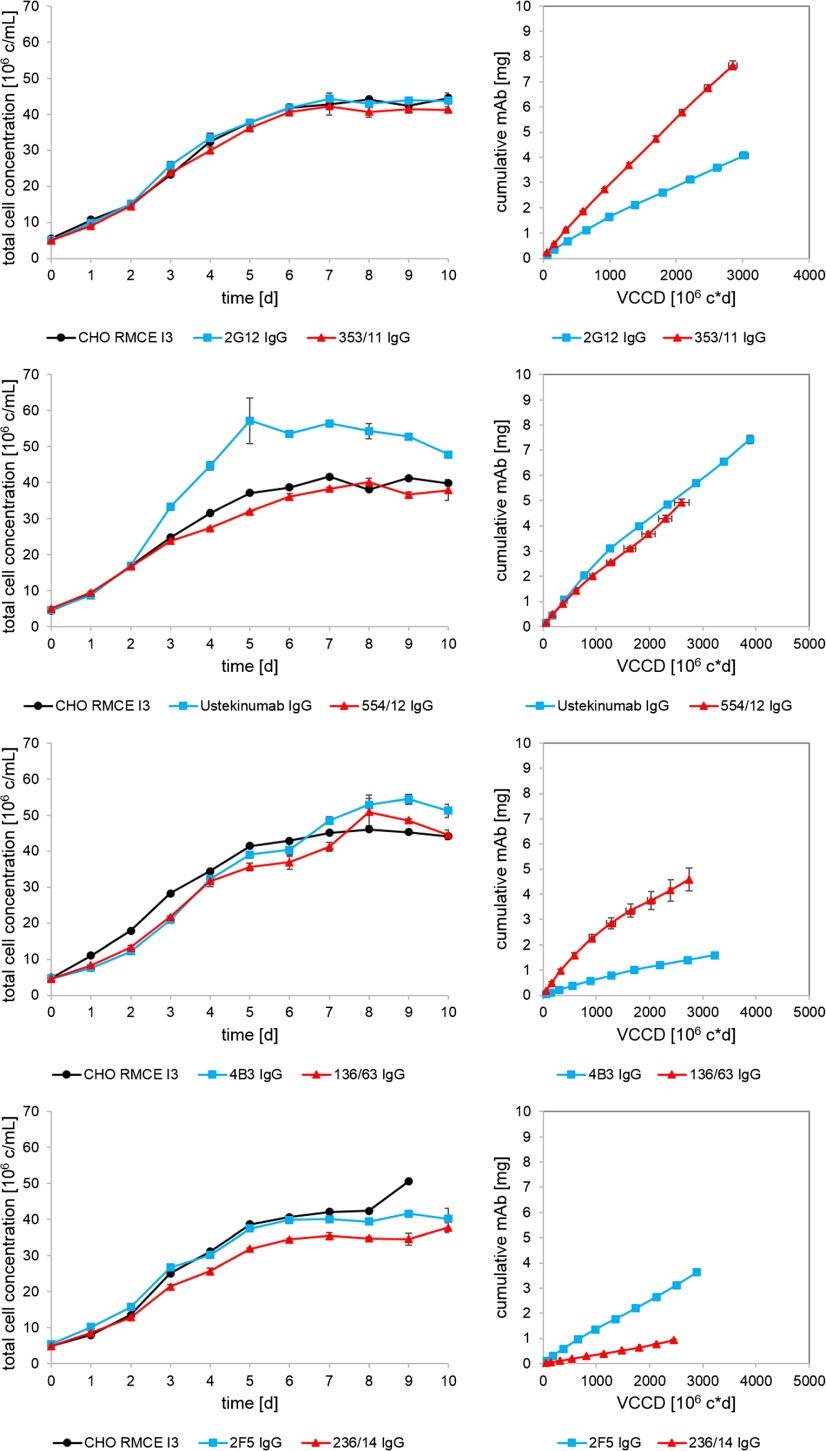
Total cell concentrations and total viable cumulative cell days (VCCD) versus cumulative mAb concentration of semi-continuous perfusion cultures: 2G12, Ustekinumab, 4B3, 2F5, and corresponding germline variants. Values represent the mean of three replicates in one single run; error bars show standard deviation. The host cell line CHO RMCE I3 control (black) is a single tube in each run

To compare mature and germline IgG-producing clones, we applied semi-continuous perfusion experiments with daily medium exchange for ten consecutive days in tube reactors. Total cell concentration and cumulative mAb concentration of four individual antibody pairs and the non-producing CHO RMCE I3 host are shown in Fig. 1. All cell lines showed a total peak cell concentration of 40–50 × 10^6^ c/mL after 6 days, except Ustekinumab which plateaued at the highest total peak cell concentration (57 × 10^6^ c/mL) with a viability of > 98%. The viabilities remained high throughout the process. Four independent cultivations of the host cell line, CHO RMCE I3, showed reproducible cell numbers and viabilities. Although the total cell concentration (TCC) and viable cumulative cell days (VCCD) of Ustekinumab were higher than its germline variant 554/12, the specific productivity (qP) was very similar at 2.8 pg/c/day, indicated by a similar slope in the VCCD versus cumulative mAb plot. The germline variants of 2G12 and 4B3 showed a significant higher cell-specific mAb production rate than the mature mAbs. For 2G12, the qP nearly doubled from 1.9 to 3.4 pg/c/day in 353/11. The germinalization of 4B3 even increased the qP from 0.7 to 3.1 pg/c/day. The germline variant of 2F5 showed reduced qP (0.4 pg/c/day) compared to the mature antibody (1.7 pg/c/day).

Germinalization of 2G12 and 4B3 led to increased expression, whereas Ustekinumab already showed high germline-like expression levels and 236/14 showed major expression issues. Moreover, our data indicated that mAb clones, 353/11, 136/63, 554/12, and Ustekinumab performed well, while 2G12, 2F5, 4B3, and 236/14 had a diminished production capacity, which was subsequently investigated in detail by identifying possible cellular or biophysical liabilities.

### Western blot of intracellular and secreted product and flow cytometry

**Fig. 2:**
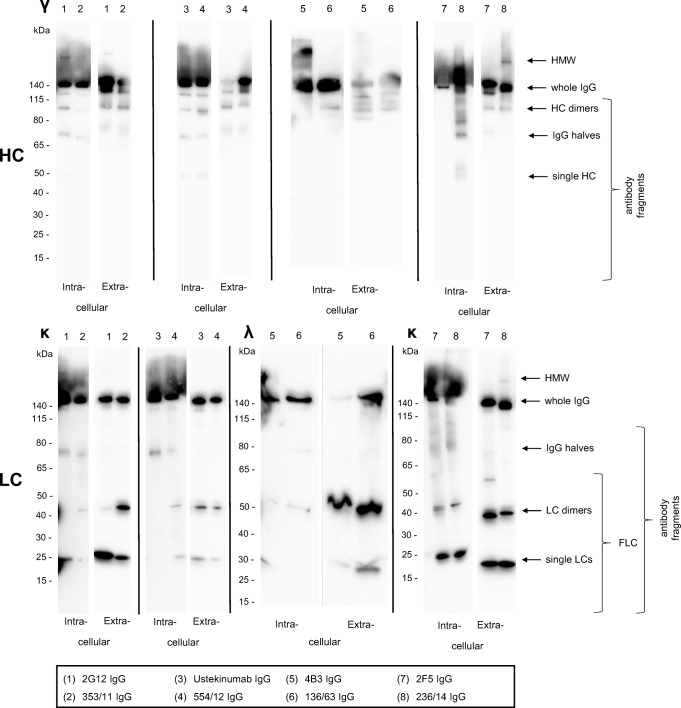
Western blots of whole cell lysates (intracellular IgG) and secreted IgG antibodies separated by SDS-PAGE under non-reducing conditions. The cell lysate and the culture supernatant were processed individually for antibody heavy or light chains. Intracellular antibody fragments of the RMCE cell lines were evaluated after 4 days in cultivation and extracellular IgG fragments were examined from the semi-continuous perfusion supernatant pool. Antibody fractions (high-molecular-weight species (HMW), whole IgG, and antibody fragments) are indicated on the right

Electrophoretic profiling by Western blotting (WB) was used to identify possible expression bottlenecks attributable to issues in either HC or LC expression and assembly, or a combination thereof (Xu et al. 2019). Structural assessment by WB identifies intra- and extracellular presence of high-molecular-weight (HMW) aggregates, whole IgG or antibody fragments including heavy-chain dimers, IgG halves, and dimeric or single free light chains (FLC) (Fig. 2). Flow cytometric analysis of all cell lines indicated single and normally distributed histogram peaks, characteristic for homogeneous cell populations and clonal stability (Fig. S3, S4).

Individual mAbs showed a quite complex product distribution pattern in WB which was often distinguishable between intracellular protein preparation and extracellular culture supernatant (Fig. 2). However, when comparing the WB with the specific productivities (qP), the patterns become more interpretable.

Extensive intracellular single LCs were detectable in low-performing clones expressing 2G12, 2F5, and 236/14. Intracellular LC dimers were predominantly visible in 2F5 and 236/14. Intracellular distinct bands of HMW aggregates were found in 2G12 and 236/14. The lowest producing clone 236/14 showed extensive amounts of intracellular HC fragments and a double band at 50 kDa, presumably consisting of single HC and additional unknown entities. Intracellular HC accumulation for 236/14 over process time was also confirmed by sequential flow cytometry (Fig. S3). In contrast, the germline mAbs with highest qP including 353/11 followed by 136/63 and 554/12 showed intensive bands for the intracellular whole IgG molecule with only minor amounts of intracellular HC fragments or aggregates (Fig. 2). High expression of the best germline mAbs was supported by lowest amounts of intracellular FLCs.

The interpretation of the extracellular product distribution leads to the conclusion that it does not give additional information on cellular productivity since the secreted protein has passed the cellular control mechanism already. Quality evaluation in the culture supernatant is most important to describe the raw material for downstream processing. Based on the summarized results, we conclude that high expression of mAbs correlates with a low fraction of intracellular FLCs and low amounts of intracellular high- or low-molecular-weight heavy-chain species.

### Endoplasmic reticulum stress

**Fig. 3:**
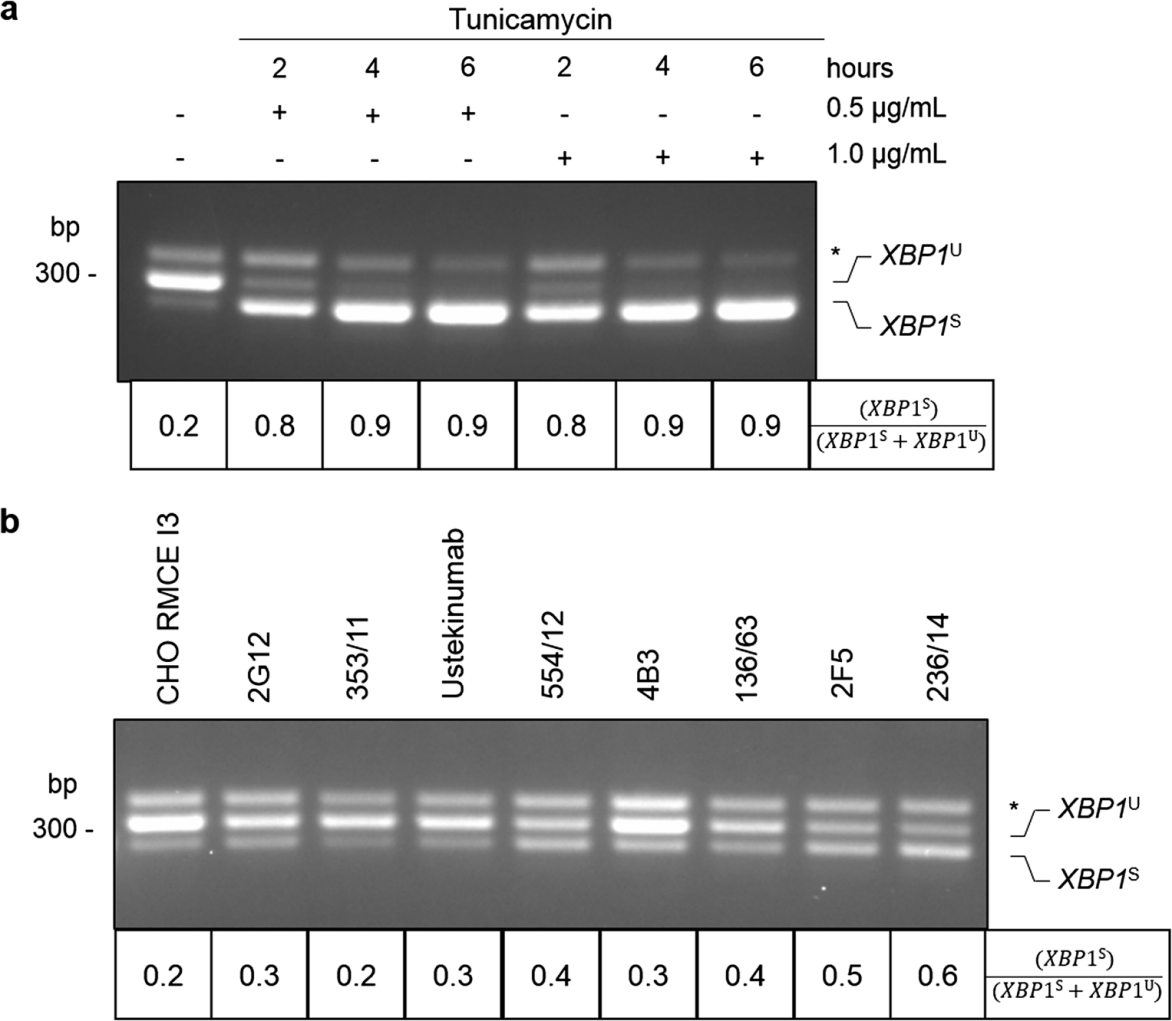
Unfolded protein response (UPR) in CHO cells. PCR product of *XBP1* corresponds to unspliced (*XBP1*^U^), spliced (*XBP1*^S^), and a hybrid product (Shang and Lehrman 2004), which is marked with an asterisk. **a** As controls, CHO K1 cells are shown untreated and treated with a gradient of tunicamycin (TM). **b***XBP1* splicing of CHO RMCE I3 and antibody-producing cell lines (2G12, Ustekinumab, 4B3, 2F5, and corresponding germline variants). The extent of IRE1α activation is indicated by the ratio of (*XBP1*^S^)/(*XBP1*^S^ + *XBP1*^U^)

To investigate the potential cellular stress response due to recombinant mAb expression, *XBP1* splicing was used as a marker for ER stress. ER stress is caused by the accumulation of unfolded proteins in the lumen of the ER and triggers activation of the UPR. There are three UPR signaling pathways, which are mediated by IRE1, ATF6, and PERK. Upon activation of IRE1, *XBP1* mRNA is spliced and translated into a potent transcription factor that upregulates expression of chaperones and components of the degradation machinery (Ron and Walter 2007). Figure 3a shows *XBP1* splicing in CHO K1 cells where untreated cells serve as an unstressed negative control. To induce ER stress, CHO K1 cells were treated with two concentrations of the ER stress-inducing drug, tunicamycin (TM, 0.5 μg/mL and 1 μg/mL), and harvested at three time points (2 h, 4 h, and 6 h). Addition of TM results in a severe increase of the spliced *XBP1*, *XBP1*^S^, compared to the unspliced *XBP1*, *XBP1*^U^. We estimated the degree of UPR by calculating the ratio of *XBP1*^S^/(*XBP1*^S^ + *XBP1*^U^). Figure 3b shows *XBP1* splicing in recombinant CHO cell lines compared to the host cell line CHO RMCE I3. All recombinant CHO cell lines reveal lower ER stress levels relative to the TM-treated cells. 2F5 and 236/14 indicate highest *XBP1*^S^ mRNA and notably, both variants resulted in low specific productivities in the semi-continuous perfusion experiment (Fig. 1). However, only minor differences between all antibody variants were observed compared to the TM-treated control, and furthermore the levels of stress did not correlate with antibody production for all clones. Although the lowest expressing mAb 236/14 also showed highest *XBP1* splicing, we conclude that ER stress monitored by *XBP1* splicing is unlikely to contribute solely to the observed difference in antibody secretion.

### Endothermic transition

**Fig. 4:**
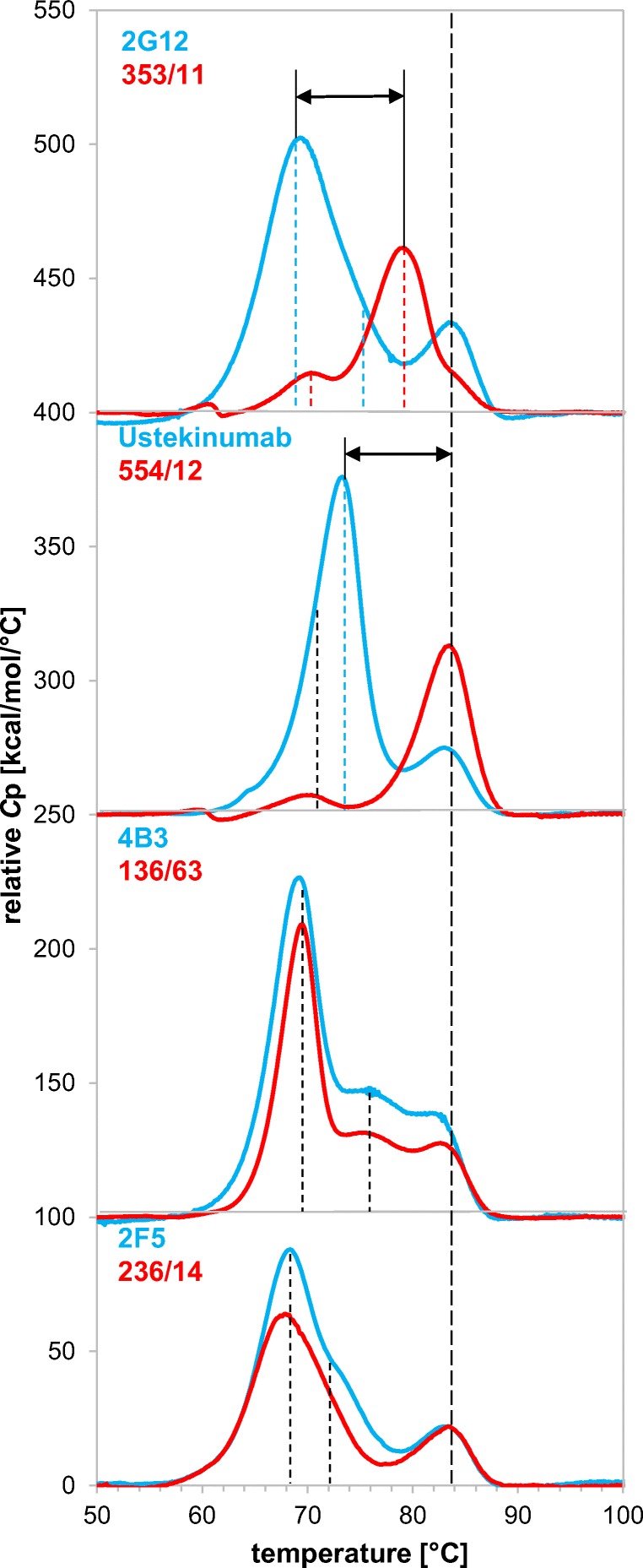
Differential scanning calorimetry (DSC) scan of 2G12, Ustekinumab, 4B3, 2F5, and corresponding germline variants. Vertical dashed lines mark the respective endothermic transition midpoints. Black arrows indicate the transition difference of mAb pairs

Differential scanning calorimetry (DSC) measurements allow direct evaluation of intrinsic thermal properties of mAbs and enable the comparison of endothermal transition temperatures for the IgG sample set. We applied DSC to all purified mAbs and evaluated thermal stability as a critical feature for assessing conformational integrity of biotherapeutics or difficult-to-express proteins. Typically, three endothermal unfolding peaks are expected for a typical human IgG1: fragment antigen binding (Fab), CH2 and CH3 domain in which the cooperative Fab unfolding shows a three-times-higher peak maximum compared to CH2 or CH3. However, it was also reported that the Fab might unfold non-cooperatively resulting in two transition peaks that may overlap with the CH2 or CH3 transition (Garber and Demarest 2007). All of the eight IgG variants revealed three irreversible unfolding transitions and showed no notable structural instabilities (Fig. 4) indicating conformational integrity within the typical range reported in literature (Jain et al. 2017). The melting temperature (*T*_m_) of the CH3 domain was comparable throughout all variants in the range of 82–84 °C and *T*_m_ of CH2 was found between 68 and 71 °C. The Fab transition can be identified by the maximum heat capacity (*C*p_max_) values, which are approximately threefold greater than the unfolding of CH2 or CH3. Observed small peaks in the pre-transition baseline at about 60 °C of the 353/11 and 554/12 are artifacts which were also found in the buffer baseline.

Figure 4 shows that the Fab unfolding transition of the germline variant of 2G12, 353/11, is increased. Similarly, the germline variant of Ustekinumab, 554/12, exhibits a drastic increase of the thermal stability. The melting temperature of the germline variant of 4B3 was comparable to the mature antibody variant 4B3. Fab *T*_m_ values of 236/14 were similar compared to its mature variant 2F5. Interestingly, slight differences of unfolding transitions of the CH2 and the CH3 domains were also observed. The Fab unfolding can be not solely represented by the Fab peak, but can also partly unfold with the CH2 or CH3 domain. This explains minor changes in *T*_m_ of CH2 and CH3 despite identical constant regions of mAbs. Thermal stabilities of Ustekinumab and its germline variant are higher than the *T*_m_ values of the other model antibodies.

### Hydropathy and aggregation potential of mAbs

An important quality attribute for recombinant proteins is solubility and therefore we aimed to identify insoluble protein aggregate formation. Analysis of the hydropathy on the Kyte-Doolittle scale shows the distribution of hydrophilic and hydrophobic residues throughout the variable sequence from their amino acid sequences (Fig. S5) (Kyte and Doolittle 1982). Here, the V_H_ reveals more deviations between the mature and the germline antibody sequence than the V_L_. Particularly, the variable heavy chains of 2G12 and 353/11 differ in their hydropathy. As Ustekinumab exhibits the highest germline identity of the chosen antibody set (Table 1), only minor changes in the CDR-H1 and CDR-H2 are observed (Fig. S5: CDRs are marked in gray). The V_H_ of 4B3 shows a striking difference to 136/63 in the FR3/CDR-H3 transition, where 4B3 displays a region with high hydrophobicity. Conversely, the germline variant 236/14 compared to 2F5 exhibits a higher hydrophobicity in the CDR-H3 loop. Model antibodies 4B3, 2F5, and 236/14 revealed hydrophobic maximum near CDR-H3 (Fig. S5). Notably, these mAbs showed the lowest specific productivities. Hydropathic indices of the variable light chains were very similar and show only minor changes near the CDR-L3 loop.

Comparison of aggregation-prone regions of the paratopes was performed by the SAP. The SAP allows a detailed view on aggregation-prone patches and identifies potentially critical sites for protein stability (Chennamsetty et al. 2009; Chennamsetty et al. 2010).

**Fig. 5:**
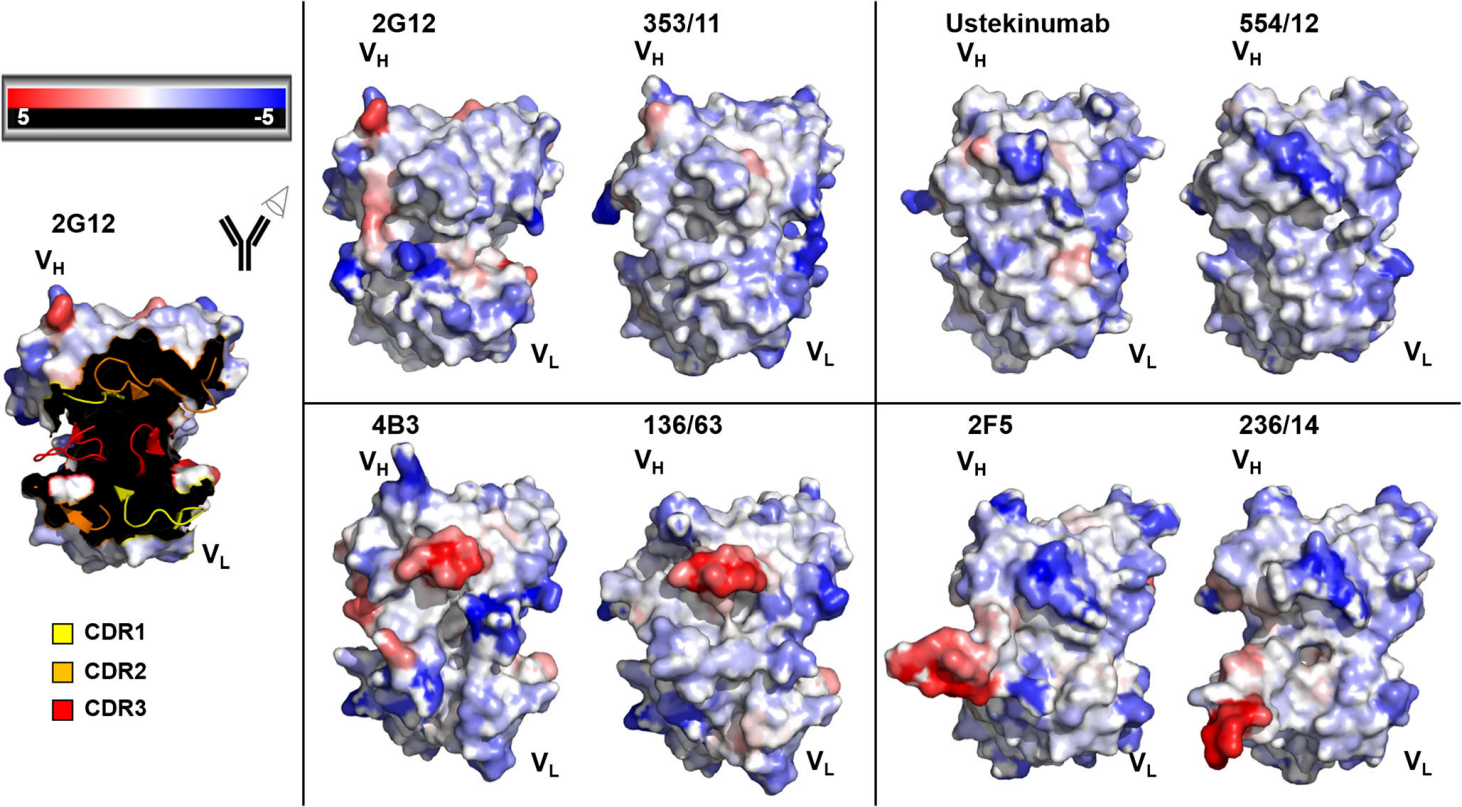
Computationally modeled V_H_ and V_L_ of 2G12, Ustekinumab, 4B3, 2F5, and their corresponding germline variants using PIGSPro (Marcatili et al. 2008). Prediction of antibody aggregation-prone regions is based on “spatial aggregation propensity” (SAP) (Chennamsetty et al. 2009; Chennamsetty et al. 2010) and visualized using PyMOL Molecular Graphics System, version 1.7.4.5., Schrödinger, LLC. The surface model is colored by SAP values: red is high SAP, blue is low SAP, and white is neutral. V_H_ is represented in the upper left area of the paratope, whereas V_L_ is in the lower right part. On the left, in the example of 2G12, CDR loops are indicated as cartoon (CDR1 = yellow, CDR2 = orange, CDR3 = red) and framework regions are represented as surface

The SAP of the variable regions of 2G12, Ustekinumab, 4B3, 2F5, and their corresponding germline variants reveals differences and similarities (Fig. 5). More regions with high SAP values, which indicate aggregation-prone regions, are observed in 2G12 compared to 353/11. The differences in the HC FR3 region of 2G12, L74, are particularly striking. Ustekinumab shows a similar SAP pattern compared to 554/12, although the germline variant exhibits the distribution of the lowest SAP values of all eight variants. Both 4B3 and 136/63 show an aggregation-prone CDR-H2 loop (Fig. 5), whereas the long CDR-H3 loop of 2F5 and 236/14 seems to have an unfavorable contribution to the antibody stability.

### Expression potential of stable IgG CHO clones versus scFv-Fc HEK293 transient transfection pools

Transient gene expression as a complementary strategy to monoclonal recombinant cell lines allows not only the comparison between stable CHO cell lines and transient HEK293 transfection pools, but also full-length IgG versus scFv-Fc antibody format. Multiple individual transient transfections were performed for each variant to ensure that minor fluctuations, arising from the transfection procedure, can be neglected. Figure S6 shows the differences in peak growth rate, which seem negligible compared to the tremendous difference in peak qP. The germline variants of 2G12 scFv-Fc, Ustekinumab scFv-Fc, and 4B3 scFv-Fc show a significant improvement of the expression potential. However, like in the IgG format, 2F5 scFv-Fc exhibits a better expressability compared to its germline variant. Significantly, the trends of the higher and lower production rate are consistent throughout the homogenous stable cell lines and the heterogeneous cell pools.

## Discussion

It is commonly accepted that only a modest number of recombinant production clones is able to produce high amounts of mAbs, e.g., above 5 g/L (Shukla et al. 2017). The reason is challenging to investigate since different factors contribute to the formation of an optimal expression cell line and the production process influences the output of protein expression. Finally, the genetic construct including the expressed transgene itself determines the expression potential of the cell line.

In this study, we evaluated if changes in the amino acid sequence of mAbs that accumulated during antibody maturation can be correlated with the expression potential of mAbs and their intrinsic thermostability. Therefore, we selected human monoclonal antibodies and designed for each of it a so-called germline variant by combining the most related germline V(D)J segments. This means that all deviations of the mature mAbs are back-mutated to germline-derived amino acids. The model antibodies were the human anti-HIV1 mAbs 2G12, 4B3, and 2F5 exhibiting an outstanding number of somatic mutations and Ustekinumab comprising low germinality compared to other human therapeutic antibodies. Host cell line, expression vector, integration locus, and gene copy number were kept constant. This allowed comparison of the expression potential under isogenic conditions at identical cultivation conditions. As a cultivation process, we chose a semi-continuous perfusion to supply the cells daily with nutrients and avoid accumulation of toxic by-products. The different antibodies showed distinct differences in mAb productivities (Fig. 1), which was in agreement with the specific productivities in routine and batch cultures (data not shown). In previous studies, it was suggested that the expression potential is primarily driven by the choice of the V_H_ gene family. Particularly, the V_H_3 family results in the highest productivity and stability compared to all other V_H_ families (Ewert et al. 2003; Ewert et al. 2004; Igawa et al. 2011; Ling et al. 2018). Besides, it can be expected that the germline families V_H_1, V_H_3, and V_H_5 result in better expression and stability properties than V_H_2, V_H_4, and V_H_6. Indeed, we observed favorable expression of the variants belonging to uneven germline families, 353/11, 136/63, and 554/12, which was also reported by Ewert et al. (2003).

Analysis of ER stress revealed only minor differences in the ratio of spliced *XBP1* (Fig. 3). One of the poorest expressing variants including 2F5 and its germline 236/14 showed the strongest signals for spliced *XBP1*. For the latter variant, this might be attributed to intracellular accumulation of heavy-chain fragments (Fig. 2) which is known to induce the UPR (Bhoskar et al. 2013). However, only the IRE1-*XBP1* branch was analyzed in our study, and therefore the remaining two branches of the UPR signaling pathway (PERK and ATF6) could be analyzed to fully understand if expression of any of these antibodies results in ER stress and whether this correlates with secretion.

Another aspect is the amount of expressed LC, generally assumed to contribute preferable to mAb expression (Bhoskar et al. 2013; Pybus et al. 2014). Underrepresentation of LCs might lead to the accumulation and aggregation of free intracellular HCs in so-called Russell bodies (Stoops et al. 2012). Our flow cytometry data indicate that the intracellular HC content remains constant over the perfusion process meaning no accumulation of probably unassembled HC. Conversely, the intracellular LC signal in flow cytometry slightly increases in the course of the process, but this cannot be assigned to an increase of free light chains in the WB signal. The occurrence of free LC dimers can be explained by distinct conserved amino acids of the LCs (Y36, Q38, and Y87), which are involved in interdomain hydrogen bond formation (Schiffer 1996). Only 2G12 does not feature all these amino acids and shows reduced LC dimer formation compared to 353/11.

Stability variation between IgG samples of the same isotype is most often defined by differences in melting temperature of the Fab fragment, as CH2 and CH3 thermal transition points are highly conserved. In previous studies, we observed that the thermal stability of IgM antibodies was improved by increasing the germinality (Chromikova et al. 2015). Clark et al. (2006) highlighted that the amino acid composition in the antibody-antigen interface is often decreased in tyrosine, serine, and tryptophan residues, but increased in histidine, proline, and phenylalanine in the course of the maturation process (Clark et al. 2006). Both applies for our mAb couples (Table S1) and further leads to the assumption that H, P, and F decrease conformational flexibility and thereby improve binding affinity of mature mAbs (Kuroda et al. 2012) (Fig. S7). Julian et al. (2017) demonstrate that such destabilizing factors of the variable region requires compensatory mutations in order to maintain thermal stability which again leads to reduced germinality of mature mAbs (Julian et al. 2017). As previously assumed, the germline variants of 2G12 and Ustekinumab showed significantly higher thermal stability of the respective Fabs, whereas the germline variants of 4B3 and 2F5 had nearly identical transition temperatures as the mature mAbs. Furthermore, an inverse correlation of DSC Fab stabilities with peak hydropathic indices of the CDRs was found, but the observed expression potential does not always correlate with thermal stability.

Besides conformational stability, colloidal stability is a relevant measure and should be taken into consideration for the design and selection of a promising antibody candidate (Goldberg et al. 2011; Geng et al. 2014). Colloidal stability can influence expression yields as well as other biophysical properties (Perchiacca et al. 2011; Dudgeon et al. 2013). Therefore, we predicted the spatial aggregation potential (Chennamsetty et al. 2009) of the mAbs by analyzing the dynamic exposure of hydrophobic patches and evaluated if this tool correlates with expression potential. Particularly, CDR-H2 loop of 4B3 and 136/63 and CDR-H3 loop of 2F5 and 236/14 were identified to be aggregation prone (Fig. 5). Those regions were comparable for mature and germline variants and both pairs did not show differences in the DSC measurements (Fig. 4). The lower expressing mature mAbs 2G12 and 4B3 show a slightly increased SAP in CDR-H3 compared to their germline variants. Similarly, the germline 236/14 shows a slightly increased SAP compared to 2F5, in agreement with the higher expression of the mature variant. Overall, the expressability of an individual mAb is challenging to predict by SAP analysis of the primary sequence of mAb.

To enable the testing of different mAb variants, we finally correlated the transient expression of homodimeric scFv-Fc molecules and received good correlations with the expression potential of stable cell lines. For the development of recombinant cell clones expressing a new mAb, we recommend screening of different sequence variants transiently to identify the best producing sequence for subsequent stable expression. Additionally, thermal stability of scFv-Fc variants correlated with the respective IgG variants (data not shown). Germinalization improved the expression potentials or antibody properties for the V_H_3 and V_H_1 antibody model pair, concluding that germinalization is able to improve antibody properties if the chosen germline family itself shows beneficial properties. This effect might be caused by introduction of unusual residues in course of maturation, but may also lead to beneficial alterations, as it was the case for 2F5. Nevertheless, focusing on selection of the human germline family with the best biophysical properties, the VH3 family, does not always lead to the desired result (Honegger et al. 2009). Taking this one step further, it seems not always favorable to use the closest germline family as basis for enhanced expression design. It might be hypothesized that it is beneficial to choose a similar germline with preferential properties that also occurs at least in high frequency in the natural antibody repertoire.

In conclusion, a stable RMCE host cell line and eight mature or germline IgG expressing cell lines were established. Overall, our model antibodies did not show a consistent and universal correlation between somatic mutations and mAb expression or thermal stability between all tested antibody pairs. Rather, it seems that germline variants are heterogeneous among themselves and therefore each mAb should be considered a unique case study. For 2G12 and 4B3, the germline variant showed enhanced expression, whereas Ustekinumab already shows high germline-like expression levels and the germline of 2F5 intrinsically showed major expression issues including lowest thermal Fab stability, a hydrophobic maximum in CDR-H3, and presence of intracellular single heavy-chain species. In our view, this emphasizes the complexity of the antibody expression and engineering as well as the need for extensive analytical methods to identify and finally relieve prevalent expression liabilities as highlighted for a model antibody set in this study. Understanding of the underlying cellular and biophysical properties of antibody expression should finally allow the antibody engineer to combine higher expression levels with maintained affinity, specificity, and functionality.

## Electronic supplementary material

ESM 1 (PDF 1099 kb)
