# Supplementary material for: Germinality does not necessarily define mAb expression and thermal stability
Source: Appl Microbiol Biotechnol. 2019 Jul 26;103(18):7505–18. doi: 10.1007/s00253-019-09998-3 (PMC6719414; doi:10.1007/s00253-019-09998-3)
Supplement: Supplementary file 1 — (PDF 1099 kb) [file 253_2019_9998_MOESM1_ESM.pdf]

Supplementary Material

Journal: Applied Microbiology and Biotechnology

Manuscript title:

'Germinality does not necessarily define mAb expression and thermal stability'

Authors:

Linda Schwaigerlehner[1], Patrick Mayrhofer[1c], Matthias Diem[2], Willibald Steinfellner[1], Emma Fenech[3], Chris Oostenbrink[2], Renate Kunert[1]

Affiliations:

[1] Department of Biotechnology, [2] Department of Material Sciences and Process Engineering, University of Natural Resources and Life Sciences, Muthgasse 18, 1190 Vienna, Austria

[3] Ludwig Institute for Cancer Research, University of Oxford, Oxford, UK

[c] Corresponding author: patrick.mayrhofer@boku.ac.at; *+43 1 47654-79861*

Content

Amino acid alignments of V_H and V_L of mature antibodies 2

Establishment of the host cell line CHO RMCE I3 4

Cultivation of isogenic IgG-producing cell lines 5

Flow cytometric analysis of intracellular HC and LC 5

Hydropathic index of V_H and V_L 7

Comparison of IgG and scFv-Fc antibodies in stable CHO cell lines and transient transfections 8

Specificity mature and germline antibodies 9

References 11

Amino acid alignments of V_H and V_L of mature antibodies

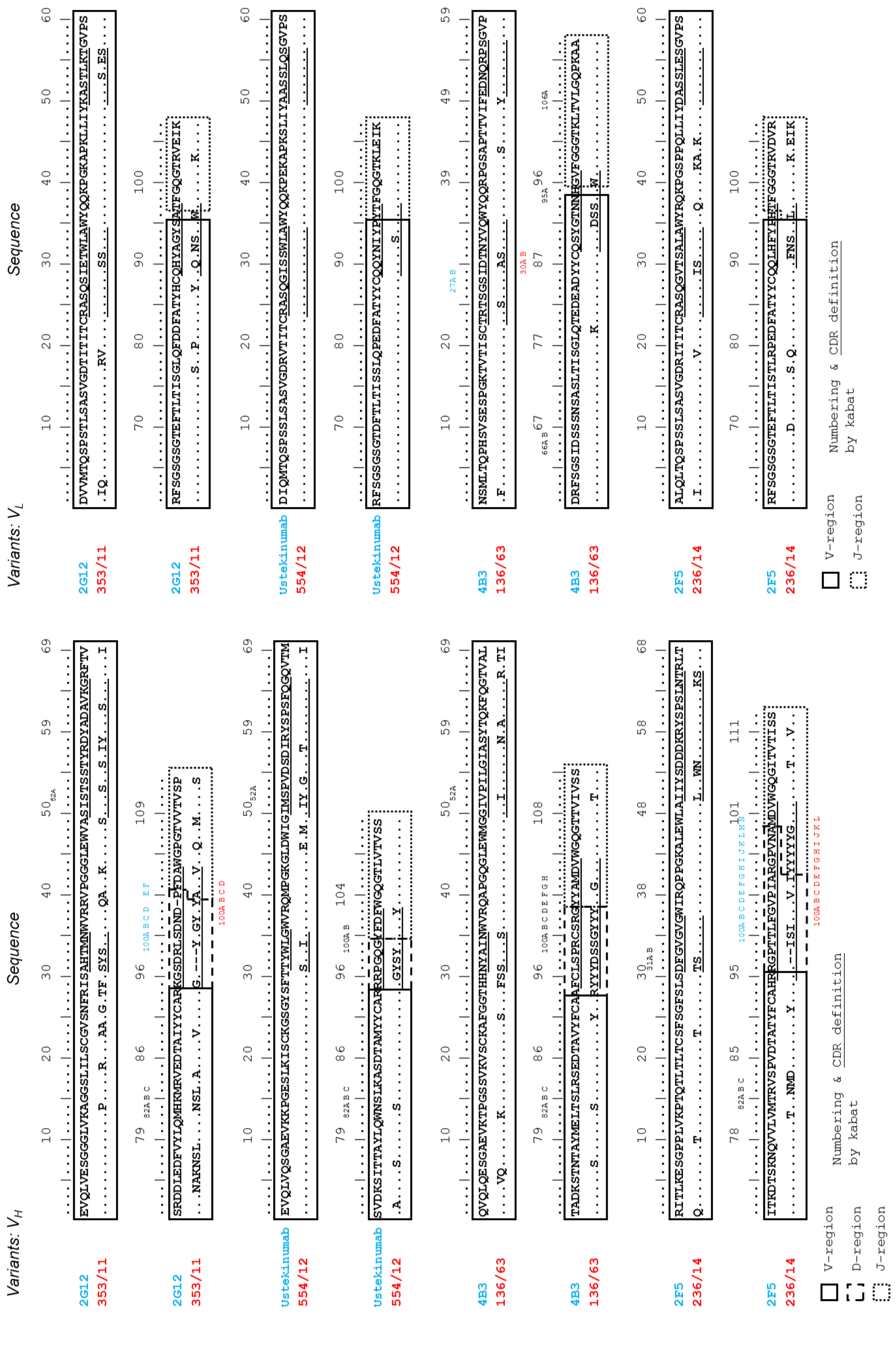

Figure S1: Amino acid alignments of V_H and V_L of mature antibodies (2G12, Ustekinumab, 4B3 and 2F5) to their nearest related germline variants (353/11, 554/12, 136/63, 236/14). Numbering and CDRs were defined by Kabat using the abYsis tool (Kabat *et al.*, 1991; Swindells *et al.*, 2017). V(D)J regions were determined by IMGT/DomainGapAlign (Lefranc, 2014) and alignments of D-regions were performed by EMBOSS (Rice *et al.*, 2000).

Establishment of the host cell line CHO RMCE I3

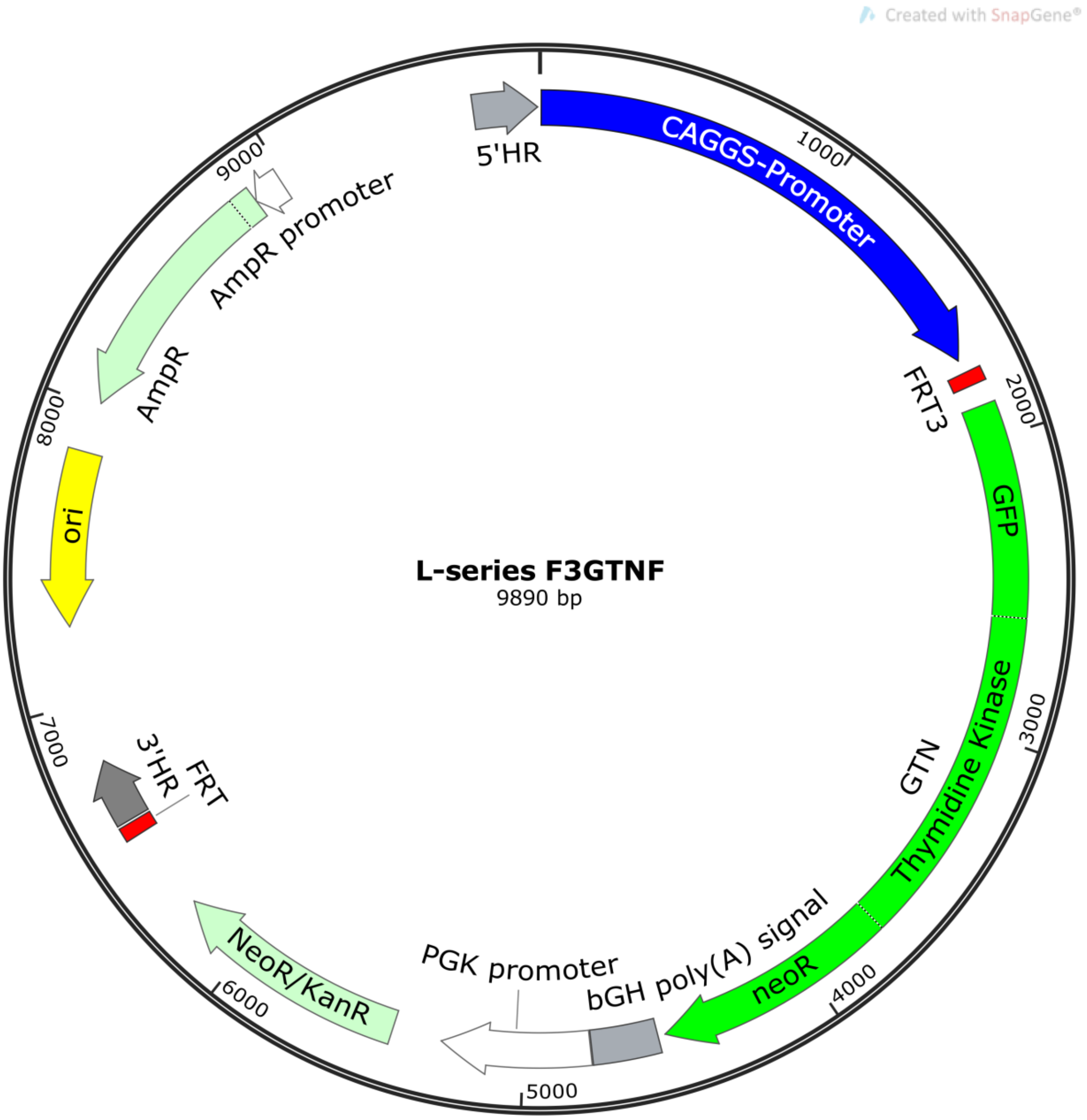

Figure S2: Plasmid map for host cell line development of CHO RMCE I3. To establish a recombinase-mediated cassette exchange (RMCE) system in CHO K1 cells (ATCC CCL-61) a CAGGS promoter was placed 5' upstream of the flippase recognition target (FRT) site to form a promoter trap in the parental RMCE cassette. The fusion protein GTN (gfp/thymidine-kinase/neomycin-phosphotransferase) serves as positive and negative selection marker and

includes the reporter GFP. GTN is located between the two heterospecific FRT sites to be exchanged during the integration of the target gene.

Cultivation of isogenic IgG-producing cell lines

Established CHO cell lines were routinely cultivated in CD CHO supplemented with 4 mM glutamine, 15 mg/mL phenol red and 2 µM GCV in 125 mL Erlenmeyer shake flasks (Corning) at a working volume of 24 mL. Cultivation was done in an ISF1-X incubator (Kuhner) operated at 37°C, 140 rpm, 7% CO_2 and 90% relative humidity. They were seeded at a cell concentration of $2*10^5$ c/mL and split every three to four days.

Flow cytometric analysis of intracellular HC and LC

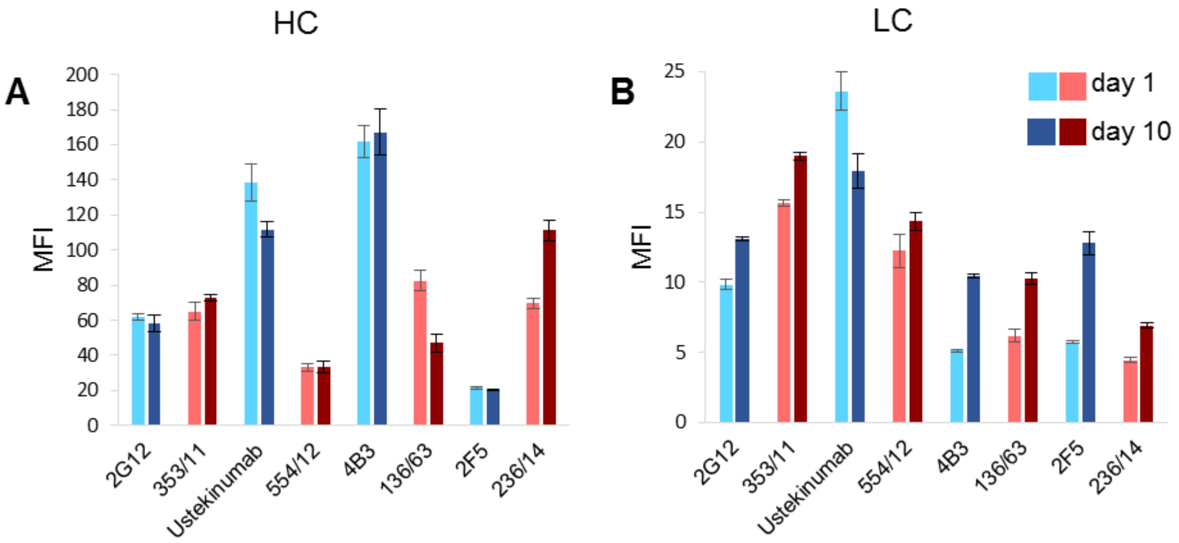

Figure S3: Flow cytometric analysis of intracellular heavy (A) and light chain (B) of eight RMCE cell lines in the semi-continuous perfusion experiment. Samples were taken on day 1 and day 10. CHO RMCE I3 host cell line was used as negative control. Mean values of

intracellular heavy or light chain of IgG producing clones are shown by median fluorescence intensities (MFI).

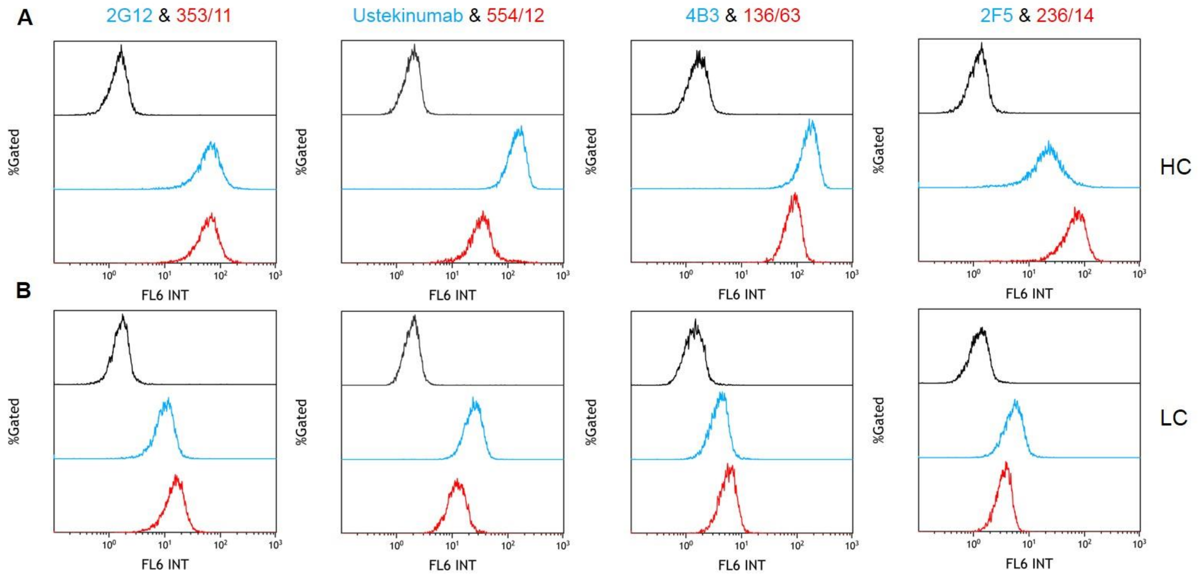

Figure S4: Histograms of flow cytometric analysis of intracellular (A) heavy chain and (B) light chain of eight RMCE cell lines in semi-continous perfusion. Samples of mature (blue) and germline (red) antibodies.

Hydropathic index of V_H and V_L

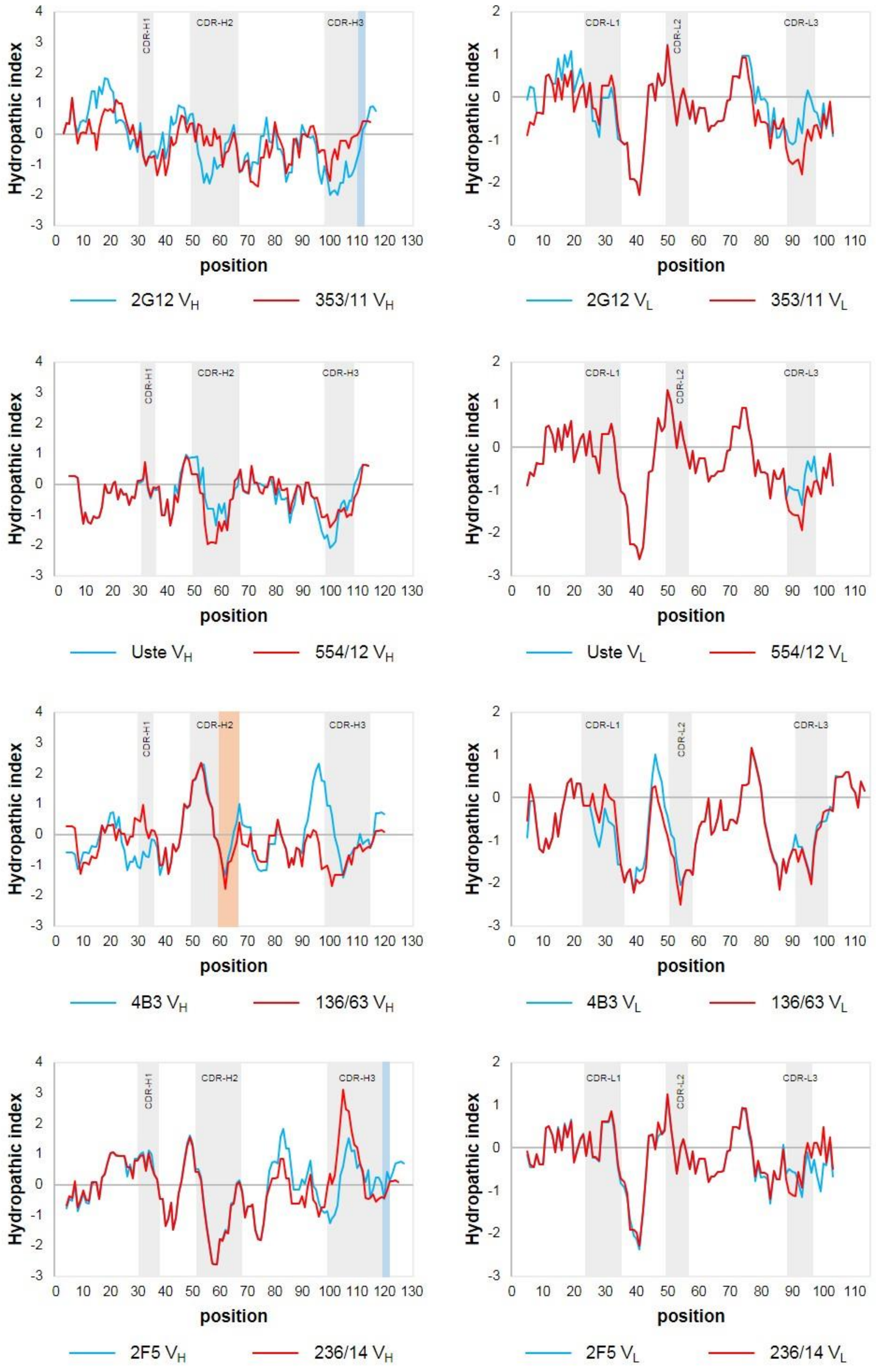

Figure S5: Kyte-Doolittle hydropathy plot of V_H and V_L (Kyte and Doolittle, 1982). Kyte-Doolittle analysis using ProtScale (http://web.expasy.org/protscale/) (Gasteiger *et al.*, 2005). Parameters were set to: window size: 9, relative weight: 100%, linear model, no normalization. The CDRs are marked in grey for the mature and the germline variant. Residues within the CDRs of only the mature variant are shown in light blue and of the germline variant in light red.

Comparison of IgG and scFv-Fc antibodies in stable CHO cell lines and transient transfections

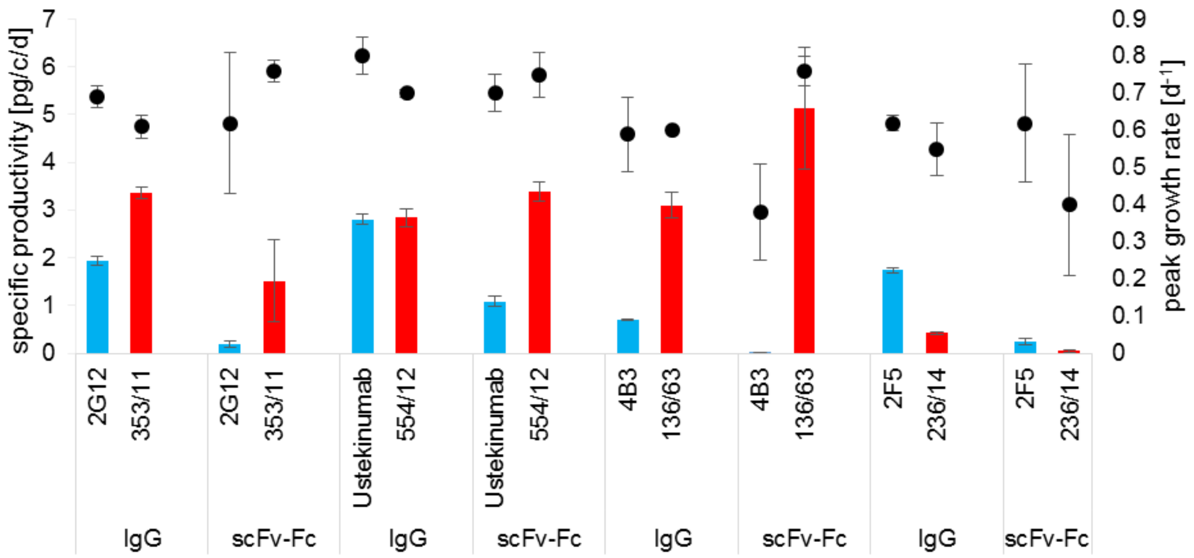

Figure S6: Peak growth rate and peak specific productivity of IgG and scFv-Fc antibodies. IgG's were produced in semi-continuous perfusion experiment with stable CHO cell lines. Same mAb variants were expressed in the scFv-Fc format in HEK293 transient transfection cell pools. Values represent the mean of at least three replicates. Black dots indicate peak growth rate.

Specificity mature and germline antibodies

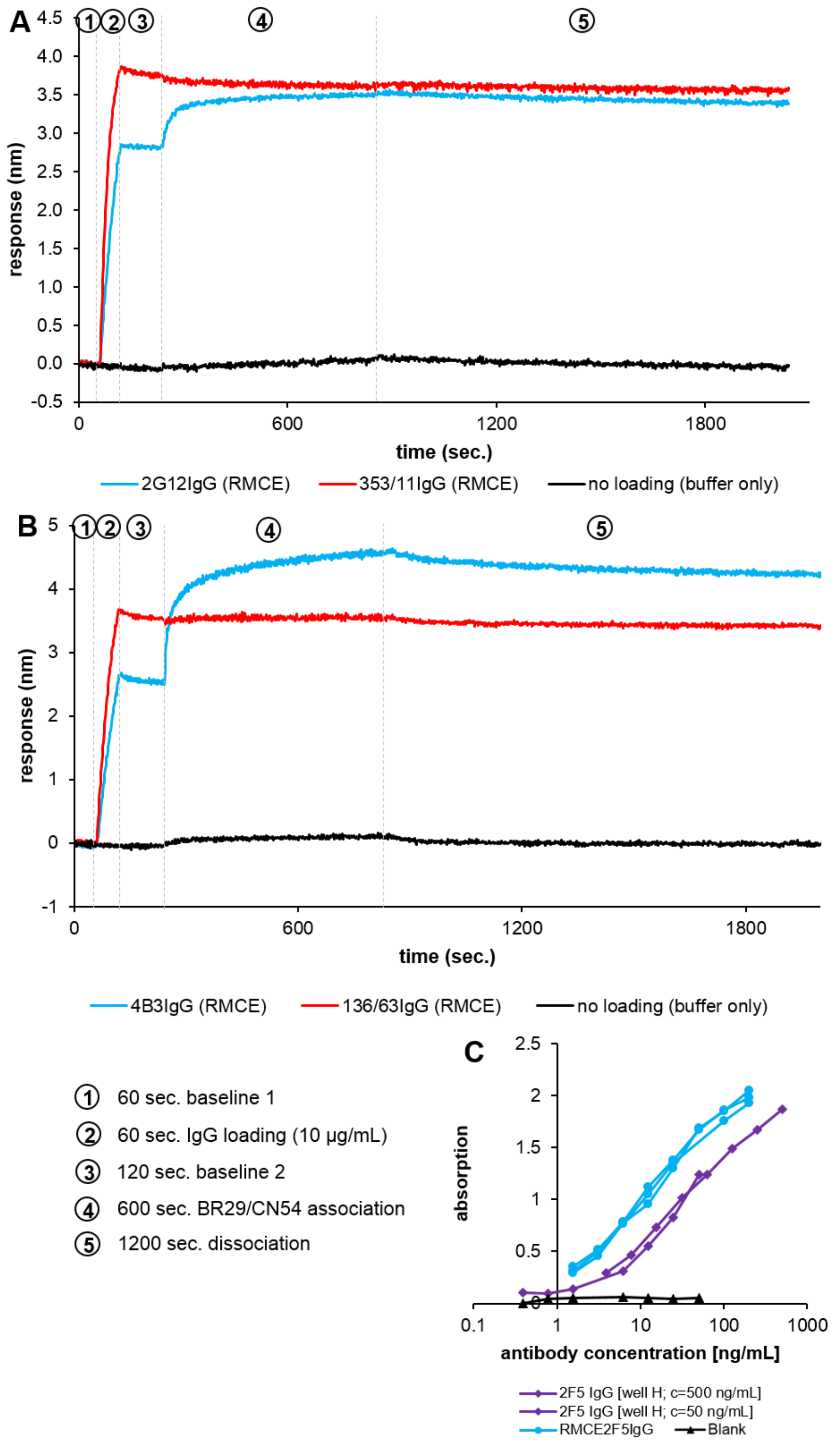

Figure S7: Evaluation of specificity of (A) 2G12 & 353/11 to BR29 (188 µg/mL), (B) 4B3 & 136/63 to CN54 (368 µg/mL) and (C) 2F5 IgG to UG37.

Table S1: Number of selected amino acids in variable region of mature and germline antibody variants. Histidine, proline and phenylalanine is increased in mature variants and tyrosine, serine and tryptophan is increased in germline variants as described in Clark *et al.*, 2006.

	increased in mature variant			increased in germline variant		
**V_H & V_L**	**H**	**P**	**F**	**Y**	**S**	**W**
2G12	4	8	9	10	26	5
353/11	0	7	8	16	40	6
Ustekinumab	0	11	8	14	33	7
554/12	0	10	7	18	37	7
4B3	4	11	7	11	30	4
136/63	2	10	5	19	39	5
2F5	3	17	10	8	28	4
236/14	1	12	8	14	30	5

References

Clark,L.A., Ganesan,S., Papp,S. and Vlijmen,H.W.T. van (2006) *J. Immunol.*, **177**, 333–340.

Gasteiger,E., Hoogland,C., Gattiker,A., Duvaud,S., Wilkins,M.R., Appel,R.D. and Bairoch,A. (2005) In Walker,J.M. (ed), *The Proteomics Protocols Handbook*. pp. 571–607.

Kabat,E.A., Wu,T. Te, Perry,H.M., Gottesman,K.S. and Foeller,C. (1991) Sequences of Proteins of Immunological Interest U.S. Department of Health and Human Services, National Institutes for Health, Bethesda, MD.

Kyte,J. and Doolittle,R.F. (1982) *J.Mol.Biol.*, **157**, 105–132.

Lefranc,M.P. (2014) IMGT® Immunoglobulin Repertoire Analysis and Antibody Humanization Elsevier Ltd.

Rice,P., Longden,L. and Bleasby,A. (2000) *Trends Genet.*, **16**, 276–277.

Swindells,M.B., Porter,C.T., Couch,M., Hurst,J., Abhinandan,K.R., Nielsen,J.H., Macindoe,G., Hetherington,J. and Martin,A.C.R. (2017) *J. Mol. Biol.*, **429**, 356–364.
